# Evaluation of mental health of students in healthcare education programs at Qatar University

**DOI:** 10.12688/mep.20055.1

**Published:** 2024-05-21

**Authors:** Ola Hayk, Abdulla Mansoor, Shahd Al-Najdi, Alaa Daud, Rula Shami, Najah Al-Hashimi, Kamran Ali

**Affiliations:** 1 QU Health College of Dental Medicine, Qatar University, Doha, 2713, Qatar; 2 Dentistry, Hamad Medical Corporation, Doha, Doha, Qatar

**Keywords:** Anxiety; Depression; Healthcare students, Mental health, Stress, Education

## Abstract

**Introduction:**

Mental health issues among undergraduate health-care students are a growing concern. This research aims to explore the frequency of mental health issues among health-care students in medicine, dentistry, pharmacy, nutrition, biomedical sciences, nursing, and public health at Qatar University.

**Methods:**

Ethics approval was obtained from the institutional review board. A total of 1,378 health-care students were invited to participate. Data were collected online using two validated questionnaires including the Patient Health Questionnaire (PHQ-9) to assess symptoms of depression, and Depression, Anxiety, and Stress Scale (DASS-21), and two open-ended questions investigating risk factors and recommendations for enhancing institutional support.

**Results:**

A total of 270 health-care students completed the survey; 227 female, and 43 male students. According to PHQ-9 cut-off values, 37.7% of students had mild depression symptoms, 25.5% moderate, 14.8% moderately severe and 10% severe symptoms. DASS-21 responses revealed 34.7% displayed severe to extremely severe anxiety symptoms, 15.4% severe to extremely severe stress symptoms and 21% severe to extremely severe depression symptoms. Students aged 18–21 years had significantly higher depression (p=0.03) and stress scores (p=0.05). Qatari students had significantly higher anxiety scores (p=0.05). Responses to open-ended questions were categorized into sub-themes and grouped together into broader themes. Most students reported exam stress and workload as key factors contributing to their negative mental health. Participants’ recommendations included reducing academic workload through better curricular planning, providing training to faculty to better support students with mental health issues, and improving mental health services.

**Conclusion:**

This study showed a significant percentage of respondents reported symptoms of stress, anxiety, and depression during undergraduate studies. Participants represent the future healthcare force for the country and there is a need to identify and support students with mental health issues through close monitoring, and work with all stakeholders to improve student support services.

## Introduction

Mental health issues among students pursuing healthcare education have gained increasing recognition in recent years. Globally, the prevalence of mental health problems among healthcare students has been mounting, raising concerns about their overall well-being. According to the World Health Organization (WHO), health encompasses not only physical well-being but also mental and social well-being ( https://www.who.int/data/gho/data/themes/theme-details/GHO/mental-health). Mental health is defined as "the capacity to feel, think, and act in ways that enhance the enjoyment of life and the ability to deal with challenges" ^1^. Viewed through this lens, mental health encompasses eight dimensions which reflect the cognitive, emotional, and interpersonal skills that are essential for human development and adaptation: attention, memory, executive functions, communication, emotion regulation, empathy, cognitive flexibility, and social functioning ^2^.

In healthcare education, mental health plays a pivotal role in determining the success and resilience of future healthcare professionals. Recent research has highlighted a myriad of factors that may contribute to poor mental health among healthcare students, including curriculum overload, assessment stress, and fear of failure, practical workload, patient care, and internships abroad. Furthermore, the learning environment, poor social life, faculty-student relationship, and financial burden have all been identified as potential stressors affecting the mental health of these students ^3^. The multifaceted dimensions of poor mental health can lead to stress, anxiety, depression, and other issues that can impair students’ academic performance, professional efficacy, sleep quality, eating habits, and daily routine ^4^. It has also been reported that mental health can also affect the physical health and psycho-emotional well-being of students ^5^.

Studies have indicated a significant association between mental health issues, such as stress, anxiety, and depression, and their adverse impact on both academic performance and personal well-being ^6, 7^. These findings underscore the need for proactive measures to address mental health challenges among healthcare students. Another challenge associated with poor mental health is social stigma. Many studies have shown that “people with mental health issues” (PWMI) are often perceived as possessed or dangerous, despite the advances in psychological support and medicine. As a result, they face avoidance and discrimination from their own communities. This stigma can affect the mental health of healthcare professionals, who may experience stress, burnout, and isolation ^8, 9^. It can also lead to a delay in seeking treatment, which can have potentially serious consequences such as life-threatening events ^10^.

Qatar has recognized mental health as a priority area in its healthcare services and has expanded it to primary care settings due to the increased demand in the country ^11– 13^. However, there is limited research on the mental health issues of healthcare students in Qatar, particularly Qatar University, who represent the future workforce and leadership of the Qatari healthcare system. A cross-sectional study by Mahgoub *et al.* (2022) reported a high prevalence of depression, anxiety, and psychological distress symptoms among medical students in Qatar ^14^ The study also revealed that stigma was a significant barrier for seeking help and support for mental health issues among 31.9% of the students. Such results highlight the importance of exploring the mental health status and needs of all healthcare students. Further research is warranted to identify the proportion of students with mental health issues to support the future healthcare workforce in Qatar and ensuring the sustainability of the country's healthcare services.

The aim of this study was to evaluate the mental health of students enrolled in healthcare education programs at Qatar University.

## Methods

### Research ethics

Ethical approval was obtained from the institutional review board at Qatar University (Approval Number: QU-IRB1797-EA/23 January 2023). Participation was voluntary and all data were collected and processed anonymously. All participants provided consent online prior to providing their responses to the questionnaire. This was in line with the requirements of research ethics approval for this study. All participants were over 18 years of age.

### Study design and target population

 This research entails a cross-sectional approach involving quantitative data collection, utilizing online questionnaires conducted between January 2023 and May 2023 to screen for three main parameters in mental health, namely depression, anxiety, and stress.

### Sample size calculation

 Given a total of 819 registered students at Health colleges of Qatar University at the time of the study and considering response rates reported in similar surveys from literature, 95% confidence interval and 5% margin of error, the minimum sample size was estimated to be 262. To reduce the risk of any potential bias from the sampling process, all registered students at QU Health Colleges were invited to participate.

### Sampling technique

 A non-probability sampling technique was employed to recruit students from Health Cluster colleges including 500 students from the College of Medicine, 110 students from the College of Medicine, 50 students from the College of Nursing, 164 students from the College of Pharmacy, and 549 students from the College of Public Health at Qatar University.

Inclusion criteria:

- Undergraduate students enrolled in medicine, dentistry, pharmacy, nutrition, nursing, biomedical sciences, and public health at Qatar University.
- Above 18 years old

Exclusion criteria:

- Students who have interrupted their studies
- Students from colleges other than healthcare colleges

### Data collection instruments

The target participants were contacted through their institutional email addresses and invited to participate in an online survey. The department of student affairs acted as the gatekeeper for sending the invites. A reminder was sent two weeks later.

The survey questionnaire consisted of five structured sections and was administered online using Google Forms. The full questionnaire is available with the underlying data for this study.

The first section included 9 questions obtaining informed consent from participants to ensure they are above 18 years-old, read the participation sheet, know who to contact in case of any confusion, understood that the participation is voluntary, and that the data will be processed confidentiality.

The second section included 6 items related to demographics to collect information on the following variables: age, gender, nationality, college, year of study and financial support.

The third section included all questions from the patient health questionnaire (PHQ-9) which is a validated, well-established, multipurpose instrument available in different languages for screening, diagnosing, monitoring, and measuring the severity of depression ^15^. PHQ-9 is a 9-item scale derived from the full PHQ. Five levels of depression disorder severity are represented by the PHQ-9 score as follows: none (0–4), mild (5–9), moderate (10–14), moderately severe (15–19), and severe (20–27).

The fourth section included the Depression, Anxiety and Stress Scale (DASS21). DASS-21 has demonstrated satisfactory reliability, and construct validity ^16^. The DASS-21 is the short-form version of the original self-reported 42-item questionnaire. It includes three self-reported scales designed to measure the negative emotional states of depression, anxiety and stress. Each of the three scales contain 7 items scored on a Likert scale from 0–3 (0: Did not apply to me at all, 1: Applied to me to some degree or some of the time, 2: Applied to me to a considerable degree or a good part of the time, 3: Applied to me very much or most of the time). The cut-off points for anxiety were 0–7 (normal), 8-9 (mild), 10–14 (moderate), 15–19 (severe) and >20 (extremely severe). The cut-off points for stress were 0–14 (normal), 15–18 (mild), 19–25 (moderate), 26–33 (severe) and >34 (extremely severe). The cut-off for depression were 0–9 (normal), 10–13 (mild), 14–20 (moderate), 21–27 (severe) and > 28 (extremely severe).

The last section included open-ended questions targeting participants with self-perceived mental health issues. These questions were aimed at exploring the key factors affecting the mental health of the participants and how the university could support them better in managing their mental health.

### Data analyses

All analyses were carried out using SPSS version 28.0 (IBM New York USA). Data analyses are presented as means and standard deviations for continuous variables (student scores) and frequency distributions for categorical variables. Normality of continuous variables was visually assessed using histograms, and by investigating skewness and kurtosis parameters. A bivariate analysis using One-way Analysis of Variance test (ANOVA) was used to assess the association between the demographic characteristics and each of the overall scores of PHQ-9 and the 3 DASS-21 subscales. Nonparametric alternative (Kruskal-Wallis test) was performed as a sensitivity analysis for any scale that showed deviation from normality. Reliability analysis was conducted by calculating Cronbach's alpha for each subscale, questionnaire, and for the entire survey. A two-sided p-value of < 0.05 was considered statistically significant.

## Results

Responses were provided by 271 participants including 229 female and 42 male students. The demographics and baseline characteristics of the participants are summarized in Table 1.

**Table 1.** Demographics and baseline characteristics of participants.

Characteristic	Distribution	N (%)
Sex	Female	229 (84.5)
	Male	42 (15.5)
Age-group	18 to 21 years	215 (79.3)
	22 to 25 years	56 (20.7)
Nationality	Qatari	104 (38.4)
	Non-Qatari	145 (53.5)
	*Missing*	22 (8.1)
Year of study	Year 1	87(32.1)
	Year 2	80 (29.5)
	Year 3	43 (15.9)
	Year 4	53 (19.6)
	Year 5	4 (1.5)
	Year 6	4 (1.5)
College	College of Dental Medicine	67 (24.7)
	College of Medicine	95 (35.1)
	College of Pharmacy	18 (6.6)
	College of Nursing	25 (9.2)
	College of Health Sciences, Biomedical Sciences	16 (5.9)
	College of Health Sciences, Public Health	5 (1.8)
	College of Health Sciences, Human Nutrition	10 (3.7)
	College of Health Sciences, Physical Therapy and Rehabilitation	35 (12.9)
Financial support	Scholarship	108 (39.9)
	Self-finance	64 (23.6)
	Sponsorship	73 (27.0)
	Other (unspecified)	26 (9.5)
	Moderate	32 (11.8)

A summary of participants’ mental health scores on PHQ-9 and DASS-21 questionnaires along with the number and percentage of participants affected is provided in Table 2.

**Table 2.** Summary of participant scores for PHQ-9 and DASS-21 questionnaires.

Scale	Severity	N (%)
PHQ-9 (Depression)	Normal	32 (11.8)
	Mild	101 (37.3)
	Moderate	69 (25.5)
	Moderately severe	40 (14.8)
	Severe	28 (10.3)
	*Missing*	1 (0.4)
DASS-21		
Anxiety	Normal	91 (33.6)
	Mild	51 (18.8)
	Moderate	29 (10.7)
	Severe	28 (10.3)
	Extremely severe	67 (24.7)
	*Missing*	5 (1.8)
Stress	Normal	156 (57.6)
	Mild	37 (13.5)
	Moderate	32 (11.8)
	Severe	23 (8.5)
	Extremely severe	19 (7.0)
	*Missing*	4 (1.5)
Depression	Normal	128 (47.2)
	Mild	43(15.9)
	Moderate	39 (14.4)
	Severe	22 (8.1)
	Extremely severe	35 (12.9)
	Missing	4 (1.5)

The mean scores of participants for different mental health conditions on PHQ-9 and DASS-21 questionnaires are summarised in Table 3.

**Table 3.** Summary of participants’ scores for mental health conditions.

	Mean(SD)	Minimum	Maximum	Severity level
Depression scores (PHQ9) *	10.98(6.18)	0	27	Moderate
Anxiety scores (DASS-21) **	6.58(5.18)	0	21	Moderate
Stress scores (DASS-21) ***	7.27(5.00)	0	21	Normal to mild
Depression scores (DASS-21) ****	6.23(5.51)	0	21	Mild to moderate

* 0–4 indicates no depressive symptoms, 5–9 mild depressive symptoms, 10–14 moderate depressive symptoms, 15–19 moderately-severe depressive symptoms, and 20–27 severe depressive symptoms ** cut-off points for anxiety: 0–3 (normal), 4-5 (mild), 6-7 (moderate), 8-9 (severe), and > 10 (extremely severe). *** cut-off points for stress: 0–7 (normal), 8-9 (mild), 10–12 (moderate), 13–16 (severe), and > 17 (extremely severe) **** cut-off for depression: 0–4 (normal), 5–6 (mild), 7–10 (moderate), 11–13 (severe) and > 14 (extremely severe)

The questionnaire showed high internal consistency with Cronbach’s alpha values being higher than 0.8 for all questionnaire subscales.

The scores of the participants on PHQ-9 and the three components of DASS-21 did not show any significant differences by sex, professional program, and year of study or type of financial support. Regarding nationality, Qatari students showed higher mean anxiety scores (7.37) on DASS-21 compared to non-Qataris (6.09) and the difference was significant (p=0.05). Age, on the other hand, showed significant interactions with PHQ-9 score and also Stress and Depression scores on DASS-21 as summarized in Table 4.

**Table 4.** Summary of participant scores by age group, gender, nationality, professional program, and financial support.

		PHQ-9 Depression N=270				DASS-21 Anxiety N=266				DASS-21 Stress N=267				DASS-21 Depression N=267			
		N	Mean	SD	P-Value	N	Mean	SD	P-Value	N	Mean	SD	P-Value	N	Mean	SD	P-Value
Age	18–21	214	11.27	6.05	0.34	211	6.72	5.1	0.37	211	7.57	5.01	0.05	211	6.6	5.55	0.03
	22–25	56	9.88	6.59		55	6.02	5.48		56	6.13	4.81		56	4.86	5.19	
Sex	Female	228	11.23	6.03	0.12	225	6.81	5.12	0.08	226	7.44	4.95	0.18	226	6.32	5.41	0.52
	Male	42	9.62	6.87		41	5.29	5.34		41	6.32	5.21		41	5.73	6.09	
Nationality	Qatari	104	11.75	6.19	0.08	103	7.37	5.23	0.05	102	7.73	5.08	0.18	103	6.54	5.82	0.44
	Non-Qatari	144	10.43	5.76		143	6.09	5.03		145	6.88	4.81		144	6.01	5.08	
Colleges	Dental Medicine	66	10.48	5.62	0.17	66	5.88	4.63	0.51	67	6.81	4.63	0.83	67	5.82	5.19	0.79
	Medicine	95	10.16	6.27		93	6.04	5.28		92	7.13	5.32		93	6.15	5.62	
	Pharmacy	18	11.94	6.88		17	7.35	5.48		17	7.88	5.37		17	6.41	6.31	
	Nursing	25	10.96	6.12		25	7.56	5.98		25	7.24	5.07		25	6	5.38	
	Biomedical Sciences	16	14.81	5.64		15	8.07	3.39		16	8.81	3.74		16	7.81	4.91	
	Public Health	5	11.6	4.33		5	7	6.32		5	6.8	2.95		5	6	6.44	
	Human Nutrition	10	13.4	6.53		10	8.4	4.69		10	8.9	5.23		10	8.7	6.44	
	Physical Therapy, and rehabilitation	35	11.11	6.65		35	7	5.74		35	7.11	5.42		34	5.91	5.63	
Financial Support	Scholarship	108	9.82	5.95	0.06	106	5.82	5.09	0.25	107	6.77	5.12	0.61	107	5.89	5.46	0.83
	Self-finance	63	11.62	6.37		63	7.03	5.6		63	7.49	5.26		64	6.67	5.88	
	Sponsorship	73	11.47	6.15		72	6.99	4.93		72	7.49	4.59		72	6.17	5.32	
	Other	25	12.84	6.39		24	7.58	5.11		24	7.96	4.95		23	6.43	5.38	

*p value ≤ 0.05 highlighted indicating statistically significant differences in mean scores between groups

### Responses to open-ended questions

A thematic analysis was undertaken for a nuanced exploration of participants’ perspectives as narrated in the responses to open-ended questions. First, the responses were collated, and reviewed several times to gain a comprehensive understanding of the content. Next, short labels were applied to code segments of text that represented specific views, experiences or recommendations. Subsequently, the codes were organized into potential themes by grouping together related codes that reflected broader patterns. Through iterative refinement, themes were reviewed, revised, and defined to accurately represent the underlying content. The key themes emerging from responses to each question are discussed below.

**Q.1 In your opinion, what are the key factors affecting your mental health adversely?**

Responses to this question were provided by 195 participants. challenges related to work-life balance were also identified by some participants. Student responses were categorized into themes and sub-themes, and the frequency was identified as shown in Table 5. Academic workload was identified as the major factor which affected the mental health of the participants and in particular the stress related to assessments and fear of failure surfaced as a key source of stress. Concerns related to personal health, academic environment, and social relationships also emerged as strong themes. Finally, uncertainty regarding career choices and difficulties in maintaining a healthy work-life balance were also reported.

**Table 5.** Perceived factors affecting the mental health of the participants adversely.

Theme	Sub-themes	Frequency *
Academic workload	• Workload related to studies and assessments • Fear of failure • Lack of time management skills to organize learning and preparation for assessments	++++
Health and well-being	• Poor physical health, including sleep, nutrition, and physical inactivity. • Concerns about body image • Grief, trauma, and past experiences	++
Academic environment	• Unrealistic expectations from faculty • Lack of a support system • Limited institutional facilities.	++
Social relationships	• Feeling isolated or neglected. • Challenges in interaction with teaching faculty	++
Uncertainty and self-doubt	• Anxiety and fear related to career choices and the future. • Financial difficulties Doubts about academic and personal performance.	+
Work-life balance	• Difficulty balancing education, social life, and personal time. • Lack of self-care. • Unable to comply with religious commitments	+

* Each “+” in the table represents 10% of responses.

**Q.2 How could the University support you better to manage your mental health?**

A total of 170 participants provided their responses to this question and the participants provided useful recommendations to improve the institutional support to students experiencing sub-optimal mental health. The participants suggested to moderate the academic workload of students and reduce weightage of high-stake end of semester assessments. Also, the need to raise awareness about mental health and improvements in counselling for students with poor mental health were recommended. Finally, participants emphasized the need for faculty training to better support students with poor mental health. These themes are summarised in Table 6.

**Table 6.** Recommendations by the participants to improve student support.

Theme	Sub-themes	Frequency*
Moderate student workload	• Decrease academic workload by better planning. • Decrease weightage of final exams.	++
Promote mental health awareness	• Organizing mental health awareness programs. • Provide more facilities and recreational activities to cope with stress.	++
Improve mental health services	• Providing free therapy sessions in accessible buildings. • Providing counsellors who have skills in languages other than Arabic.	+
Faculty Training	• Faculty need to be trained to support students with mental health issues. • Faculty need to listen more to students’ feedback. • Faculty need to be more flexible with academic assignments.	+

** Each “+” in the table represents 10% of responses.

## Discussion

### Demographics

The study included participants from multiple healthcare education programs at diverse stages of their education. Qatar University’s health cluster consists of medical, dental, pharmacy, nursing and health science colleges. Therefore, it was deemed crucial to explore students’ perception on their mental health across the multiple programs. Each curriculum within these programs differs in terms of credit hours, and hence the workload, the clinical contact with patients, the assessment strategies, and the support systems available. Accordingly, factors which may potentially influence students’ mental health may vary. For example, students in clinical training years may face additional pressures related to the hospital environment, and interactions with clinical staff, patients and their caregivers ^17^.

In the current study, it was noted that the year of study did not show any significant differences on either PHQ-9 and DASS scales, however, younger students (age range 18–21) showed significant relations with PHQ-9 score and also the stress and depression scores on DASS-21. This could be related to a lack of preparedness of students in early years to manage academic workload in a university setting, and inherent stress that comes with the demands of their field of study. This is in line with previous findings reporting higher prevalence of anxiety and depressive disorders in the younger population. t. Data from the Adult Psychiatric Morbidity Survey in the United Kingdom has revealed a rise in the incidence of common mental disorders (CMDs) among individuals aged 16 to 24, suggesting that today’s young adults have a higher likelihood of encountering mental health issues compared to preceding generations (NHS Digital, https://files.digital.nhs.uk/pdf/q/3/mental_health_and_wellbeing_in_england_full_report.pdf, 2014). In addition to physiological and hormonal changes, one of the factors that have been linked to this phenomenon could be “coping” ^18^. Coping has been described as “personality in action under stress” ^19^. The coping trait is characterized as inherent methods of reacting to alterations in the environment of any nature. 20 with several theories regarding spontaneous, subconscious, and involuntary reactions as elements of coping ^21, 22^ Support-seeking and self-controlling coping methods have been advocated as effective coping strategies. Therefore, it has been suggested that a deeper understanding of personality traits is required to produce tailored support ^23^

Regarding mental health symptoms of students enrolled on different health professional programs, the current study found no significant differences. This is contrary to findings from a previous study which reported that mental health symptoms were influenced by the type of profession ^17^. Pharmacy students showed more anxiety and depressive symptoms than dental or preclinic medical students. Other studies have shown that medical and pharmacy students showed a higher frequency of psychiatric symptoms compared to dental students ^24, 25^.). These variations may be related to differences in academic workload and stress of assessments amongst students at different institutions ^17, 26^. 

In terms of nationality, Qatari students showed significantly higher mean anxiety scores on DASS-21 compared to non-Qataris (p=0.05). A previous study in Qatar showed variations within Arab societies in their knowledge, attitude and practice towards mental illness, with Qatari nationals possessing poorer knowledge about causes of mental illness compared to non-Qatari Arabs ^24, 25, 27^. Other reasons for ethnic differences may be related to attitudes towards mental illness, and stigma associated with mental health issues causing people to suffer in silence. The World Health Organization (WHO) recognises the serious implications of stigma and has accordingly described Mental Health Stigma (MHS) as a significant impediment to the successful treatment of mental illnesses because it negatively affects individuals' inclination to seek treatment. Research conducted in Western settings has demonstrated an association between MHS, reluctance to seek assistance, and a heightened burden of symptoms. Many studies have concluded that MHS may lead to reluctance in seeking help, and an increase in the burden of symptoms ^28– 30^.

In contrast to conventional epidemiological observations concerning mental health, our study did not find a significant connection between being female and a higher prevalence of depression ^31^. Females outnumber males in healthcare programs at Qatar University and therefore, gender differences may have been more difficult to identify.

### Prevalence of mental illness symptoms

Mental health concerns have become more common amongst healthcare students and several studies have reported high prevalence of mental disorders among healthcare professional students. For instance, In Brazil, 30.6% of medical students were found to be depressed, 32.9% were anxious, and 46.1% had poor sleeping habits ^32^. Another study in the United Kingdom reported that 27% of the participants were depressed and had anxiety symptoms ^33^. In Nepal, a study on 226 medical and dental students showed that 92.92% (210 students) were stressed during the examinations ^34^. Another study from Hong Kong on 661 nursing students reported that 37.3% of the students were anxious, 41.1% were stressed, and 35.7% were depressed ^35^. Moreover, another study on healthcare students in France, highlighted the need for psychological support for students, considering the high frequency of depression among those studying medicine, dentistry, and pharmacy. The study revealed that the overall prevalence of 7-day anxiety and depressive symptoms, major Depressive Episodes, suicidal ideation, and humiliation were 55%, 23%, 26%, 19%, and 19% respectively in medical, pharmacy, and dental students. In addition, burnout was present in 42% of nonclinical students and 65% of clinical students and residents ^17^. Finally, one study conducted in China, found that Chinese medical students' mental health issues should be handled seriously as they reported that the prevalence of depression, anxiety, suicidal ideation, and eating disorders in Chinese medical students were 29%, 21%, 11%, and 2% respectively ^36^. A high prevalence of depressive symptoms was found amongst health-care students when compared to the general population in Qatar ^13, 37^. This might be explained by the challenges of the health profession programs and high stakes assessments.

### Self-reported factors affecting mental health and recommendations

Two open-ended questions were used at the end of the survey, the first to gain in-depth insight into key factors affecting students’ mental health adversely. Academic workload was identified as the major factor, in particular the stress related to assessments and fear of failure.

For healthcare students, the academic workload is considered significantly burdensome, contributing to stress, burnout, and mental health problems such as depression and anxiety ^38^. Similar to our findings, a study conducted in nine schools in the state of Florida, United States, investigating stressors facing medical students, found that the intensity and volume of the curriculum, alongside clinical duties, heighten the risk of emotional and physical exhaustion ^39^. The COVID-19 crisis has further compounded the stress and burnout experienced by health professions students, necessitating mindfulness and stress management interventions ^40^.

The academic environment is a critical factor in student mental health. Initiatives to increase staffing for counseling services and invest in mental health resources are common, but are often insufficient to address the campus-wide mental health crisis (Inside Higher Ed. https://www.insidehighered.com/news/student-success/health-wellness/2023/11/07/six-considerations-next-gen-college-student-mental). In the current study, unrealistic expectations from faculty were raised as an issue affecting the academic environment. This is in-line with other studies reporting that if students’ expectations differ from faculty expectations, students may experience anxiety ^41– 43^, concluding that congruent expectations among faculty and students may promote integrated and seamless student transitions from pre-clerkship to clerkships. Support systems were also deduced as a subtheme in this study. A previous systematic review identified a variety of interventions, including those focusing on pass/fail grading systems, mental health programs, mind-body skills programs, curriculum structure, multicomponent program reforms, and many more. However, only a few specific learning environment interventions were associated with improved emotional well-being, indicating the overall quality of the evidence was low, highlighting the need for high-quality medical education research in this field ^44^.

Participants in the current study also identified poor social relationships as a factor contributing to mental health issues. Literature also shows that students with characteristics that differ from their peers, such as minority status or low socioeconomic status, are at greater risk of social isolation and mental health problems. Quality social support is crucial, as students with lower perceived social support are at a sixfold risk of depressive symptoms. (Hefner and Eisenberg 2009 and Backhaus *et al*., 2023).

The second open ended question explored ways to prevent and manage mental health issues as reported in student’s own words. It was found that decreasing the academic workload and weighting of final assessments was the most frequent theme. Promoting mental health awareness and providing counsellors who have skills in languages other than Arabic was also proposed, suggesting the need to be more inclusive of non-Arabic speaking students. Moreover, it is crucial to recognize the role of individual traits, such as high levels of conscientiousness and ambition, which while contributing to academic success, may also predispose students to psychological distress ^45^. To mitigate these effects, developing faculty and administration awareness and training is essential. Reducing academic workload through better planning, promoting mental health awareness, and improving mental health services are also strategies that can support the well-being of healthcare students as recommended in the findings of the current study. Various mental health promotion and prevention interventions and their effectiveness have been discussed in the literature ^46^. Therefore, healthcare institutions should invest in systems that promote positive wellbeing and resilience among students ^47^. In addition resources should be allocated to elements that foster a positive learning environment, to ensure the prevention of mental illnesses and better outcomes among those with mental illness.

## Conclusion

The current study demonstrates a unique insight into healthcare professional students’ mental health in a country with a small population with high GDP along with a high number of expatriates. Academic workload exacerbated by lack of time management skills and fear of failure are the most common factors affecting students’ mental health. The high prevalence of self-reported mental illness symptoms among students was influenced by age and nationality. Effective interventions need to be implemented by healthcare institutions to support student mental health, focusing on belonging, resilience, and social connections.

## Data Availability

OSF: Mental Health Evaluation of Students in Healthcare Education. https://doi.org/10.17605/OSF.IO/MX2UG ^48^ This project contains the following underlying data: Mental Health Study -data file anonymised.xlsx OSF: Mental Health Evaluation of Students in Healthcare Education. https://doi.org/10.17605/OSF.IO/MX2UG ^48^ This project contains the following extended data: Full questionnaire Data are available under the terms of the Creative Commons Attribution 4.0 International license (CC-BY 4.0).
