# Supplementary material for: Evaluation of mental health of students in healthcare education programs at Qatar University
Source: MedEdPublish (2016). 2024 May 21;14:37. [Version 1] doi: 10.12688/mep.20055.1 (PMC11452764; doi:10.12688/mep.20055.1)
Supplement: Peer review history

# Reviewer report: Reviewer response for version 1

**DOI:** 10.21956/mep.21475.r38582

**Published:** 2024-10-04

**Authors:** Feriha Fatima Khidri

**Affiliations:** 1 Liaquat University of Medical & Health Sciences, Jamshoro, Pakistan

The article aims to explore the mental health status of healthcare students at Qatar University, by assessing depression, anxiety, and stress. It requires following points to address:

 1. In introduction do not mention website "( https://www.who.int/data/gho/data/themes/theme-details/GHO/mental-health)", rather cite in numerical order. 

 2." A cross-sectional study by Mahgoub *et al.* (2022)" ........Use uniform reference method.  

 3. What was the response rate of students?

 4. Add the folllowing study results to compare the results of depression of your study. 

 Khidri FF, Riaz H, Bhatti U, Shahani KA, Kamran Ali F, Effendi S, Rani K, Chohan MN. Physical activity, dietary habits and factors associated with depression among medical students of Sindh, Pakistan, during the COVID-19 pandemic. Psychology Research and Behavior Management. 2022 May 25:1311-23.

Have any limitations of the research been acknowledged?

No

Is the work clearly and accurately presented and does it cite the current literature?

Partly

If applicable, is the statistical analysis and its interpretation appropriate?

Yes

Are all the source data underlying the results available to ensure full reproducibility?

Yes

Is the study design appropriate and is the work technically sound?

Yes

Are the conclusions drawn adequately supported by the results?

Yes

Are sufficient details of methods and analysis provided to allow replication by others?

Yes

Reviewer Expertise:

Mental health

I confirm that I have read this submission and believe that I have an appropriate level of expertise to confirm that it is of an acceptable scientific standard.

## References

[1] : Physical Activity, Dietary Habits and Factors Associated with Depression Among Medical Students of Sindh, Pakistan, During the COVID-19 Pandemic. 10.2147/PRBM.S364540 10.2147/PRBM.S364540 PMC914861135642191

# Reviewer report: Reviewer response for version 1

**DOI:** 10.21956/mep.21475.r37581

**Published:** 2024-06-26

**Authors:** Ulfat Bashir

**Affiliations:** 1 O, Riphah International University (Ringgold ID: 66783), Islamabad, Islamabad Capital Territory, Pakistan; 2 Riphah International University, Islamabad, Islamabad Capital Territory, Pakistan

The manuscript is novel and objective. A very well written manuscript. Please elaborate more on the methodology section detailing rationale for each statistical method used. Also, usage of association instead of causal terminology such as effect/affect is more appropriate for this study design.

 Otherwise a very valuable addition for the medical education community.

Have any limitations of the research been acknowledged?

Yes

Is the work clearly and accurately presented and does it cite the current literature?

Yes

If applicable, is the statistical analysis and its interpretation appropriate?

Yes

Are all the source data underlying the results available to ensure full reproducibility?

Yes

Is the study design appropriate and is the work technically sound?

Yes

Are the conclusions drawn adequately supported by the results?

Yes

Are sufficient details of methods and analysis provided to allow replication by others?

Partly

Reviewer Expertise:

Medical education,Orthodontics and Artificial Intelligence.

I confirm that I have read this submission and believe that I have an appropriate level of expertise to confirm that it is of an acceptable scientific standard.
